# Comparison of the Treatment Efficiency of Bone Marrow-Derived Mesenchymal Stem Cell Transplantation via Tail and Portal Veins in CCl_4_-Induced Mouse Liver Fibrosis

**DOI:** 10.1155/2016/5720413

**Published:** 2015-12-29

**Authors:** Nhung Hai Truong, Nam Hai Nguyen, Trinh Van Le, Ngoc Bich Vu, Nghia Huynh, Thanh Van Nguyen, Huy Minh Le, Ngoc Kim Phan, Phuc Van Pham

**Affiliations:** ^1^Laboratory of Stem cell Research and Application, University of Science, VNU-HCM, Ho Chi Minh City 700000, Vietnam; ^2^Biology Faculty, University of Science, VNU-HCM, Ho Chi Minh City 700000, Vietnam; ^3^University of Medicine and Pharmacy Ho Chi Minh City, Ho Chi Minh City 700000, Vietnam; ^4^Nguyen Tat Thanh University, Ho Chi Minh City, Vietnam

## Abstract

Because of self-renewal, strong proliferation *in vitro*, abundant sources for isolation, and a high differentiation capacity, mesenchymal stem cells are suggested to be potentially therapeutic for liver fibrosis/cirrhosis. In this study, we evaluated the treatment effects of mouse bone marrow-derived mesenchymal stem cells (BM-MSCs) on mouse liver cirrhosis induced by carbon tetrachloride. Portal and tail vein transplantations were examined to evaluate the effects of different injection routes on the liver cirrhosis model at 21 days after transplantation. BM-MSCs transplantation reduced aspartate aminotransferase/alanine aminotransferase levels at 21 days after injection. Furthermore, BM-MSCs induced positive changes in serum bilirubin and albumin and downregulated expression of integrins (600- to 7000-fold), transforming growth factor, and procollagen-*α*1 compared with the control group. Interestingly, both injection routes ameliorated inflammation and liver cirrhosis scores. All mice in treatment groups had reduced inflammation scores and no cirrhosis. In conclusion, transplantation of BM-MSCs via tail or portal veins ameliorates liver cirrhosis in mice. Notably, there were no differences in treatment effects between tail and portal vein administrations. In consideration of safety, we suggest transfusion of bone marrow-derived mesenchymal stem cells via a peripheral vein as a potential method for liver fibrosis treatment.

## 1. Introduction

Epidemiological analysis has revealed an increase in deaths caused by liver cirrhosis from 676,000 to over 1 million in 2010 [1]. It has been claimed that cirrhosis affects about a hundred million people worldwide [2] and is common in sub-Saharan Africa and Asia where the rates of hepatitis B and C virus infections are high [3]. Currently, hepatic cirrhosis is characterized by accumulation of extracellular matrix (ECM) proteins [2, 4], loss of liver functions, and activation of hepatic stellate cells (HSCs) [5]. Liver dysfunction and fibrous tissue may be caused by viruses, toxins, and autoimmune, cholestatic, alcoholic liver, and metabolic diseases [2]. For liver cirrhosis treatment, orthotopic liver transplantation (OLT) is considered as the gold standard [6, 7], but there is an organ donor shortage and a large number of liver cirrhosis patients. Over the last decade, the number of OLT procedures has decreased because of the lack of organ donors [2]. In addition, lifelong immunosuppression is a considerable issue after OLT. Hence, cell transplantation is recommended as a potential approach for treatment of hepatic fibrosis. As potentially therapeutic cells, mesenchymal stem cells (MSCs) exhibit self-renewal [8, 9] and strong proliferation* in vitro* [8–10] and have abundant sources for isolation [2, 6, 11, 12]. Additionally, MSCs can be differentiated into a variety of cell types including hepatocytes [13–18].

Bone marrow (BM) consists of hematopoietic stem cells and MSCs [9]. In the early 1970s, Friedenstein et al. first described BM-MSCs [19]. Subsequently, numerous reports revealed the plasticity, high proliferation, and differentiation capacity of these cells. The properties of BM-MSCs suggest a potential for use in liver cirrhosis treatment. BM-MSCs possess many advantages such as autologous sources, abundance of cells, an immune-modulatory capacity [6, 7, 13], secretion of cytokines and growth factors [2, 6], homing to injury sites, and hepatic differentiation [2, 13, 20]. Indeed, several preclinical studies [9, 21–26] and a clinical trial [27] have been conducted to determine the effectiveness of BM-MSCs for liver cirrhosis treatment. However, there are still controversial issues including MSC engraftment, the timing and numbers of MSCs that home to the liver, and hepatic differentiation* in vivo*. The routes of MSC transplantation appear to be related to these controversies [7, 28]. In contrast, Xiang et al. [29] claimed that the route of MSC transplantation to treat liver injury induced by carbon tetrachloride (CCl_4_) did not affect the timing or number of homed MSCs. Nonetheless, the effects of different BM-MSC transplantation routes on liver cirrhosis should be examined to clarify any ambiguity. In this study, MSCs isolated from mouse BM were evaluated for their effectiveness in liver cirrhosis treatment by transplantation via the tail or portal veins. To induce a mouse model of liver cirrhosis, we used CCl_4_ that specifically damages the liver. To assess therapeutic effectiveness, we evaluated changes in liver injury/function markers, ECM gene expression, and liver histopathology combined with the Knodell or Ishak (HAI) scoring systems and fibrous protein staining [collagen type I and *α*-smooth muscle actin (*α*-SMA)]. We also determined the presence of BM-MSCs to demonstrate their homing capacity. Hence, our findings of BM-MSC transplantation routes provide a comparison of these routes and evidence of the potential of BM-MSCs for liver cirrhosis treatment.

## 2. Materials and Methods

### 2.1. Induction of Fibrosis in Mice

This study was approved by our Institutional Ethics Committee (Laboratory of Stem Cell Research and Application, University of Science, Vietnam National University). Liver fibrosis mice were induced by 1.0 mL/kg CCl_4_ according to previous publication (Truong et al., 2014) [30]. In summary, male Swiss mice were treated by 1.0 mL/kg CCl_4_ (99.5% purity, UNI-CHEM Chemical Reagent, China) via oral administration three times per week (every two days) for 11 consecutive weeks, while mice in the control group were treated with olive oil. Liver fibrosis was assessed based on the following criteria: expression of liver injury markers (serum aspartate aminotransferase (AST) and alanine aminotransferase (ALT)), liver function markers (serum bilirubin and albumin), fibrosis/cirrhosis-related genes (fibronectin, integrins, transforming growth factor-*β* (TGF-*β*), procollagen) (Table 1), anti-*α*-smooth muscle actin (*α*-SMA) and collagen type 1, and histopathology by hematoxylin and eosin (H&E) and Masson's trichrome staining.

### 2.2. Isolation, Culture, and Expansion of Mouse BM-MSCs

BM-MSCs were isolated from the femurs of healthy Swiss mice (Pasteur Institute, Ho Chi Minh City, Vietnam). BM was collected and cultured in 25 cm^2^ culture flasks containing Dulbecco's modified Eagle's medium/F12 medium supplemented with 10% fetal bovine serum and 1x antibiotic-antimycotic solution (Sigma-Aldrich, St. Louis, MO, USA). Cells were incubated at 37°C with 5% CO_2_. The medium was changed periodically after 3 days. MSCs were isolated from BM according to previous reports [8, 31]. Cells with a spindle-shaped morphology similar to fibroblasts were considered as MSC candidates. At 70–80% confluence, the cells were subcultured using 0.25% trypsin/EDTA (Sigma-Aldrich). Passages 2–5 BM-MSCs at confluence were used for analyses and transplantation.

### 2.3. Characterization of BM-MSCs

BM-MSC phenotypes were analyzed by flow cytometry using a FACSCalibur (BD Biosciences, NJ, USA). A total of 1 × 10^6^ cells/mL were incubated with FITC-, PerCP-, PE-, or APC-conjugated anti-CD34, anti-CD45, anti-CD29, anti-CD44, anti-CD90, anti-c-kit, or anti-Sca-1 antibodies (BD Biosciences) in phosphate-buffered saline (PBS) containing bovine serum albumin (BSA) for 30 minutes at room temperature.

Adipogenic differentiation was induced by culture with adipogenic medium supplemented with 10^−8 ^M dexamethasone and 10^−4 ^M L-ascorbic-acid-2-phosphate (Sigma-Aldrich, St. Louis, MO) as previously described (Ngoc et al., 2011) [32]. After 30 days of induction, cells were fixed in 3% formaldehyde in PBS for 10 minutes and stained with Oil Red O.

Osteogenic differentiation was induced by incubation with culture medium that consisted of ascorbic acid, dexamethasone, 6-glycerol phosphate (Sigma-Aldrich, St. Louis, MO), and calcium deposits which were visualized by alizarin red.

### 2.4. Transplantation of BM-MSCs

After 11 weeks of CCl_4_ administration, mouse liver cirrhosis models were divided into the following groups: Placebo-Ta: 10 mice received 0.1 mL PBS/mouse via the tail vein; Placebo-Po: 10 mice received 0.1 mL PBS/mouse via the liver portal vein; BM-MSCs-Ta: 10 mice were infused with 1 × 10^6^ cells in PBS/mouse via the tail vein; BM-MSCs-Po: 10 mice were infused with 1 × 10^6^ cells in PBS/mouse via the liver portal vein. Simultaneously, a model group (10 mice) was administrated with CCl_4_ for 11 weeks. The control group (10 mice) was treated with olive oil for 11 weeks. To confirm cell engraftment, BM-MSCs were labeled with green fluorescent protein (GFP) and then transplanted into mice.

At 3 weeks after BM-MSC administration, venous blood was obtained from the retroorbital vein to measure liver serum markers. All mice were then sacrificed for further analyses.

For tracking, we established BM-MSCs that stably expressed the* gfp* gene. We used a* cop*GFP lentiviral vector (Santa Cruz Biotechnology, Santa Cruz, CA) for transfection. Transfected cells were cultured in selection medium containing 10 *μ*g/mL puromycin dihydrochloride (Sigma-Aldrich, St. Louis, MO, USA) for 1 week to select GFP-expressing cells. The GFP expression was confirmed by fluorescent microscope and flow cytometry. We have 2 other groups (tail vein and portal vein) of liver cirrhosis mice for GFP labeled cells (3 mice/group). After 21 days of transplantation, left liver lobes were obtained to check the existence of GFP-positive cells.

### 2.5. Measurements of Liver Injury/Function Markers (Serum AST, ALT, Direct Bilirubin, and Albumin)

Venous blood was collected in 1.5 mL tubes and then centrifuged at 2000 g for 10 minutes. Plasma was obtained, and the activities of AST/ALT (Diagnosticum Zrt., Hungary) and amounts of direct bilirubin (QuantiChrom Bilirubin Assay Kit, Bioassay Systems, CA, USA) and albumin (QuantiChrom BCG Albumin Assay Kit, Bioassay Systems) were evaluated according to the manufacturers' instructions.

### 2.6. Evaluation of Fibrosis Biomarkers

Mouse liver tissues were collected, and total RNA was extracted using an Easy-BLUE Total RNA Extraction Kit (iNtrON Biotechnology, Korea) according to the manufacturer's instructions. Fibrotic gene expression was assessed by quantitative reverse-transcription polymerase chain reaction (RT-PCR) (Brilliant II QRT-PCR Master Mix Kit, 1-Step, Agilent, CA, USA) using specific primers (Table 1).

### 2.7. Immunohistochemistry

Liver tissues were obtained and fixed in 4% paraformaldehyde (Merck Millipore, Germany). Paraffin-embedded sections, which had been deparaffinized and rehydrated, were subjected to antigen retrieval in sodium citrate (10 mM, pH 6) at 95–100°C for 20 minutes. The sections were allowed to cool for 20–30 minutes at room temperature and then blocked for 30 minutes at room temperature with blocking buffer (2% goat serum, 1% BSA, 0.1% Triton X-100, 0.05% Tween 20, 0.01 M PBS, pH 7.2–7.4, and 0.05% sodium azide). Then, the sections were stained with anti-collagen type I (Santa Cruz Biotechnology Inc.) or *α*-SMA (Santa Cruz Biotechnology Inc.) antibodies at 1 : 100 dilutions in TBS/1% BSA overnight at 4°C. Cellular peroxidase was blocked with peroxidase blocking buffer (3% H_2_O_2_ in PBS) for 10 minutes at room temperature. A horseradish peroxidase-conjugated goat-anti-rabbit secondary antibody in TBS/1% BSA was applied to the sections for 1 hour at room temperature. Immunocomplexes were visualized using an ACE kit (Sigma-Aldrich) according to the manufacturer's instructions. Nuclei were stained with hematoxylin-Gill III (Merck Millipore). The sections were washed with TBS + Tween 20 two to three times between each step. The percentage of collagen-positive areas was determined by Image J software.

### 2.8. Histopathology

Liver tissues were collected and fixed in 4% paraformaldehyde (Merck Millipore), and then H&E and Masson's trichrome staining were performed. Liver tissues were collected and fixed in 4% paraformaldehyde (Merck Millipore, Germany), and then H&E and Masson's trichrome staining were performed. In H&E staining, paraffin liver section was deparaffinized by xylene, dehydrated using alcohol, and washed. Then, liver section was stained in hematoxylin (Merck Millipore, Germany) for 5 min, washed quickly, and then differentiated by 1% acid alcohol for 30 sec and washing for 10 min. Slides were stained with eosin solution (Merck Millipore, Germany) for 2-3 min, then washed and mounted.

For Masson staining, liver section was deparaffinized, dehydrated, and washed. Firstly, slides were stained with Weigert's iron hematoxylin for 5 min and washed; secondly, slides were stained with Biebrich scarlet acid fuchsin solution for 5 min and washed. Thirdly, slides were differentiated in 1% phosphomolybdic-phosphotungstic acid solution for 5 min, then transferred to aniline blue solution, and stained for 5 min. Finally, sections were differentiated in 1% acetic acid solution for 1 min, washed, dehydrated, and mounted with mounting medium.

The interpretation of results was based on the histological activity index of Knodell-Ishak (Ishak-modified HAI).

### 2.9. Statistical Analysis

Data analysis was conducted using Prism 6 software and Microsoft Excel 2011. Image J was used to analyze picture.

## 3. Results 

### 3.1. Characterization of BM-MSCs

**Figure 1:**
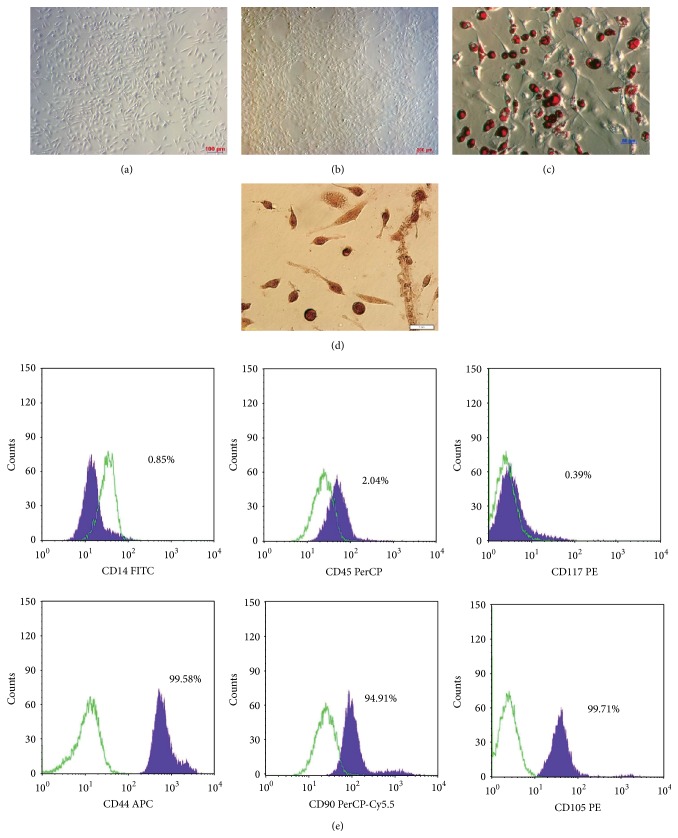
Characterization of BM-MSCs. (a) BM-MSC candidates expanded and had a fibroblastic morphology after 24 hours of primary culture. (b) MSCs reached confluency in secondary culture. (c) Differentiation of MSCs into adipogenic cells at day 30 of induction. Cells were positive for Oil red O staining. (d) MSCs were successfully differentiated into osteoblasts and were positive with alizarin red. (e) Flow cytometric analysis of passage 3 cells showed positivity for CD44, Sca-1 (Ly 6AE), and Thy 1 (CD90) and negativity for CD34, CD45, and c-Kit (CD117).

After 24 hours of primary culture, BM-MSC candidates remained spherical and attached to the culture surface. Concurrently, other cell types (blood cells and hematopoietic stem cells) were nonadherent and floating in the culture. After 72 hours, BM-MSC candidates had expanded and exhibited a spindle shape (Figure 1(a)).

At days 4–8 of culture, BM-MSC candidates proliferated strongly and reached 65–70% confluence after 2 weeks of culture. In subcultures, BM-MSC candidates maintained a fibroblast-like shape (Figure 1(b)) and were capable of colony formation at low density. MSC candidates were negative for CD34, CD45, and c-Kit (CD117) and positive for CD44, Sca-1 (Ly 6AE), and Thy 1 (CD90) (Figure 1(e)).

### 3.2. Differentiation Potential of BM-MSCs

After 72 hours of differentiation, BM-MSCs exhibited a round shape and accumulated fat droplets in their cytoplasm. Adipogenic cells appeared at day 7 of adipogenesis induction. The accumulation of fat droplets within the cells was easily observed under a microscope at ×100 or ×200 magnifications (Figures 1(c) and 1(d)). These cells were positive for Oil red O staining. This result showed that the BM-MSCs were capable of differentiation into adipocytes. Alizarin red staining showed that BM-MSCs differentiated into bone cells after 28 days of induction (Figure 1(d)).

### 3.3. Changes in Liver Injury/Function Markers after BM-MSC Transplantation

**Figure 2:**
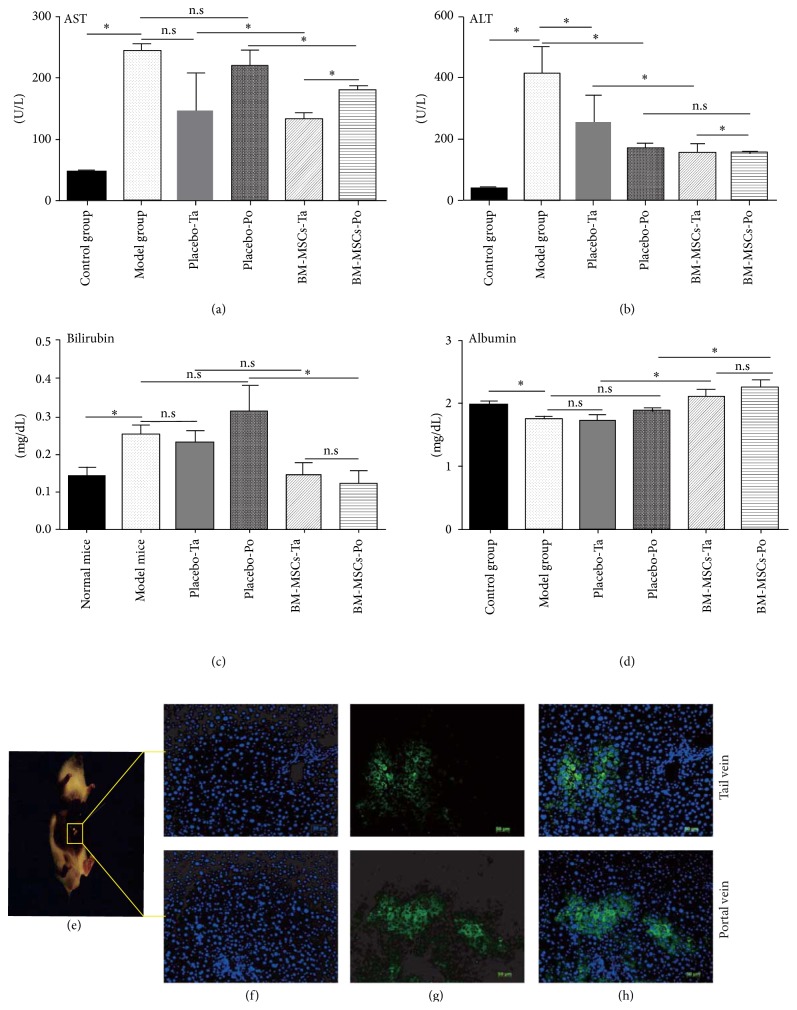
((a) and (b)) Levels of serum AST and ALT (liver injury markers) at 21 days after transplantation. ((c) and (d)) Levels of serum direct bilirubin and albumin (liver function markers). (e) GFP-positive BM-MSC graft in liver cirrhosis mice (iBox Explorer Imaging Microscope UVP, US). (f) DAPI staining. (g) GFP-postive cells (Carl Zeiss, Oberkochen, Germany) and (h) merged image. Results are the means and SD; *p* < 0.05, Student's *t*-test.

After 11 weeks of CCl_4_ treatment, both AST (242.58 ± 126.32 U/L) and ALT (412.18 ± 90.64 U/L) levels were increased dramatically by 5- and 10-fold, respectively, compared with control mice. The levels of AST and ALT in PBS-treated groups were decreased compared with those in the model group but higher than those in control mice (Figures 2(a) and 2(b)). Notably, AST and ALT levels were decreased in BM-MSC infusion groups (*p* < 0.05, compared with PBS-treated groups; Figures 2(a) and 2(b)). These results indicated that transplantation of BM-MSCs could prevent liver damage to a certain extent.

At 21 days after BM-MSC transplantation, direct bilirubin levels were decreased significantly in treatment groups. The levels of direct bilirubin were 0.145 ± 0.020 and 0.257 ± 0.0219 in control and model mice, respectively, after 11 weeks of CCl_4_ administration and continued to increase in PBS-treated groups. In contrast, the serum level of direct bilirubin decreased dramatically in BM-MSC-treated groups (*p* < 0.05, compared with PBS-treated groups; Figures 2(c) and 2(d)). A decrease in serum ALB was observed in PBS-treated groups but not in BM-MSC-treated groups (Figures 2(c) and 2(d)). These results showed that BM-MSC transplantation could prevent liver injury and facilitate liver function recovery (Table 2).

### 3.4. BM-MSC Transfusion Decreases the Expression of Fibrogenesis- and ECM-Related Genes

**Figure 3:**
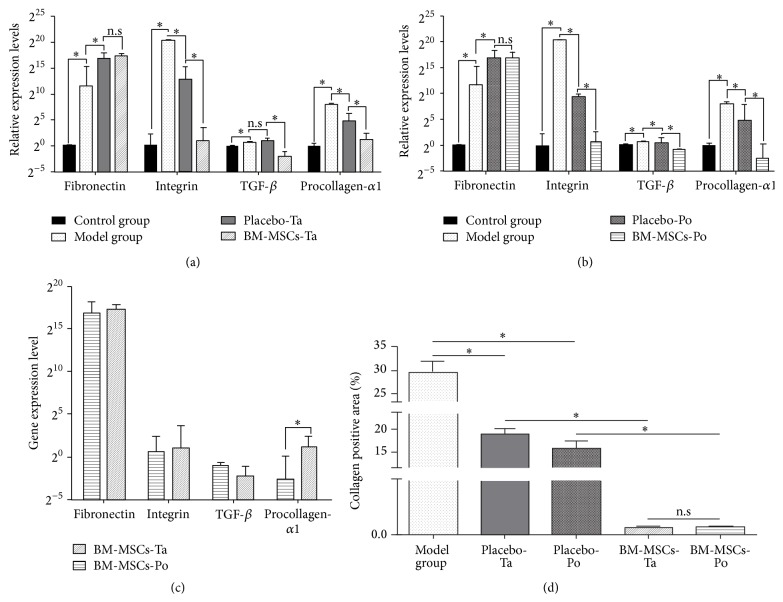
((a) and (b)) Gene expression analysis of fibronectin, integrins, TGF-*β*1, and procollagen was performed by quantitative RT-PCR. (c) Comparison of the percentages of collagen type 1-positive areas between groups. Analysis of relative gene expression data employed Livak's method (2^−ΔΔCt^). Values were normalized to the gene expression levels of the control group. *p* < 0.05, Student's *t*-test.

Quantitative RT-PCR revealed decreases in procollagen and integrin expression in PBS-treated groups (*p* < 0.05, compared with model mice, Figure 3), whereas increases in fibronectin and TGF expression were observed in PBS-treated groups after 21 days (*p* < 0.05, compared with model mice, Figures 3(a) and 3(b)). As shown in Figures 3(a) and 3(b), gene expression levels in groups that received infusion of BM-MSCs via the tail or portal veins were lower than those in PBS-treated groups. Specifically, integrin and procollagen expression was significantly reduced in treatment groups (*p* < 0.05, compared with PBS-treated mice, Figures 3(a) and 3(b)). Additionally, expression of TGF-*β* was lower in groups that received BM-MSC transplantation. Compared with PBS-treated groups, no increase in fibronectin expression was found in cell transplantation groups (*p* > 0.05). Notably, fibronectin expression was highly increased in BM-MSC-treated groups compared with the model group. However, fibronectin expression was also increased in placebo group compared to model group. There was no significant in difference in fibronectin expression between BM-MSC-and PBS-treated groups (*p* > 0.05).

In terms of inhibition of fibrogenesis-related gene expression by BM-MSC therapy, transplantation via the portal vein was more efficient than tail vein injection because a significant decrease in procollagen expression was found in BM-MSCs-Po (*p* < 0.05) but not in other groups (Figure 3(c)).

### 3.5. Antifibrotic Effects of BM-MSCs in Liver Fibrosis Mice

**Figure 4:**
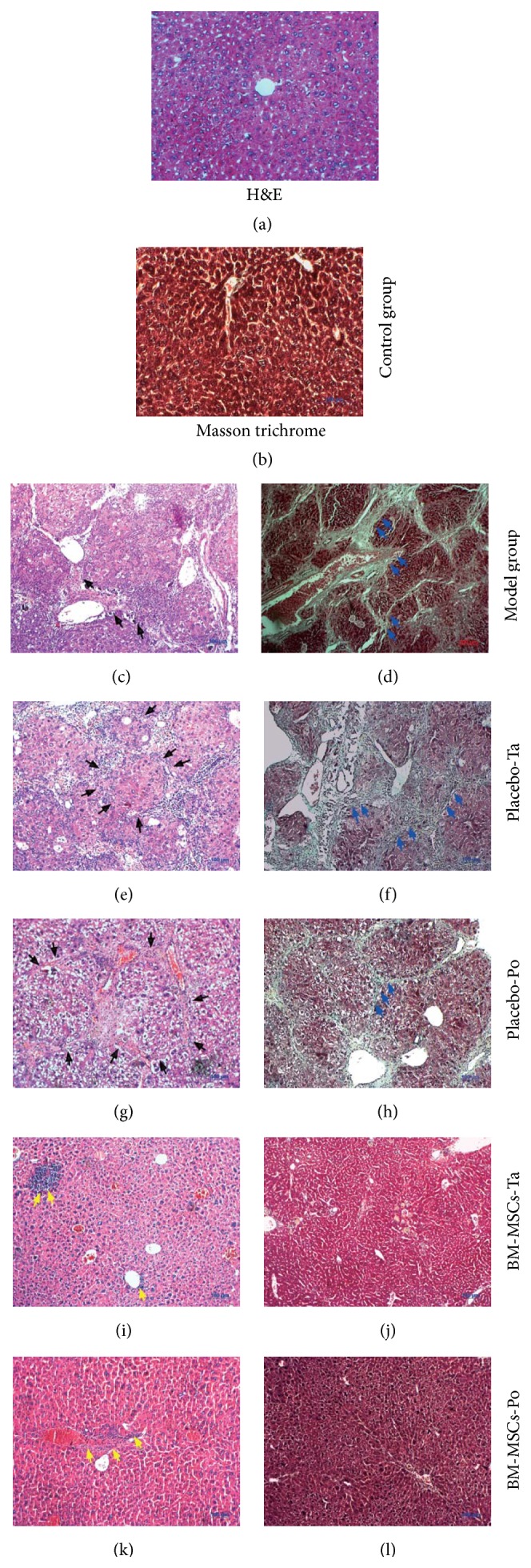
H&E and Masson's trichrome staining at 21 days after treatment. ((a) and (b)) Control group; ((c) and (d)) Model group; ((e) and (f)) Placebo-Ta group; ((g) and (h)) Placebo-Po group; ((i)-(j)) BM-MSCs-Ta group; ((k) and (l)) BM-MSCs-Po group. (a), (c), (e), (g), (i), and (k) H&E staining; (b), (d), (f), (h), (j), and (l) Masson's trichrome staining. Black arrow: pseudolobule structures adjacent to collagen fibers; blue arrow: collagen fibers; yellow arrow: inflammation area.

H&E staining showed significant changes in the structural histology between BM-MSC- and PBS-treated mice. In BM-MSC-treated groups, inflammation remained around the portal triad and central vein, but these areas were not widespread or cross connected (Figures 4(i) and 4(k)). In PBS-treated groups, there were inflammatory cells, hepatic steatosis, and necrotic cells (Figures 4(e) and 4(g)). Accompanying these characteristics, collagen fibers occupied a large area in the livers.

To further analyze liver fibrosis, we conducted Masson's trichrome staining. Collagen fibers were observed throughout the liver sections of PBS-treated and model groups (Figures 4(f) and 4(h)). Furthermore, liver tissue was divided into pseudolobule structures by these collagen fibers. In contrast, the histological structure of cell-transplanted groups had clearly fluctuated. Compared with model and PBS-treated groups, fewer fibrotic areas were found in BM-MSC-treated groups (Figures 4(j) and 4(l)). Histological grading and staging of chronic hepatitis were performed according to the Ishak-modified HAI system (Table 3). The results indicated that BM-MSC transplantation ameliorated necroinflammatory and cirrhosis scores. In particular, at 21 days after cell transplantation, cirrhosis was not observed in BM-MSC-treated mice, whereas 66.7% of mice in PBS-treated groups had a 1/6 cirrhosis score. These data showed that BM-MSC injection exerted anti-inflammation and antifibrogenic effects in liver cirrhosis mice.

**Figure 5:**
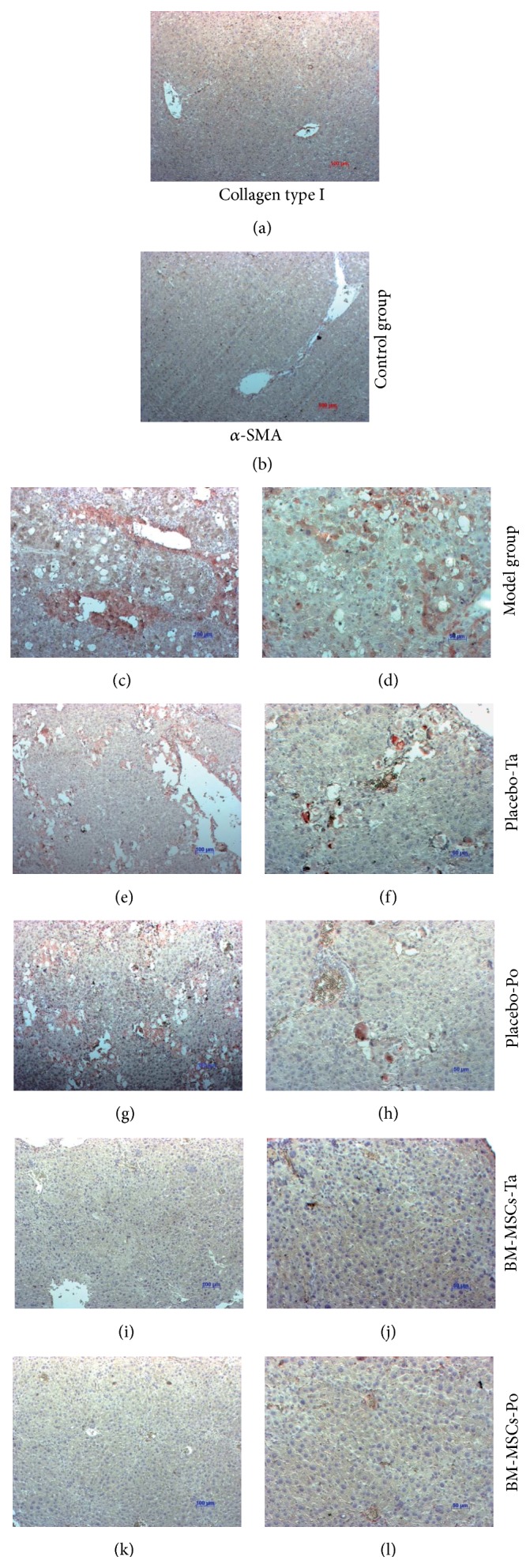
Immunohistochemical staining of collagen type 1 and *α*-SMA at 21 days after treatment. ((a) and (b)) Control group; ((c) and (d)) Model group; ((e) and (f)) Placebo-Ta group; ((g) and (h)) Placebo-Po group; ((i)-(j)) BM-MSCs-Ta group; ((k) and (l)) BM-MSCs-Po group. (a), (c), (e), (g), (i), and (k) Collagen type 1 staining; (b), (d), (f), (h), (j), and (l) *α*-SMA staining. Positive cells are red. Nuclei are blue.

Collagen type 1 and *α*-SMA staining were carried out to confirm the antifibrotic effects of BM-MSCs (Figure 5). Collagen staining was reduced in both PBS-treated groups compared with the model group (Figures 5(c), 5(e), and 5(g)). Interestingly, almost no collagen-positive area was observed in BM-MSC-treated groups (Figures 5(i) and 5(k)). The collagen staining appeared around the portal triad and central vein. In PBS-treated groups, collagen staining was distinguished in many areas of the portal triad, central vein, and central lobule. In BM-MSC transplantation groups, the percentage of the area positive for collagen type 1 was reduced significantly from 18.93 ± 1.09% to 0.137 ± 0.015% in BM-MSCs-Ta (Figure 3(d)). Similarly, BM-MSC transplantation via the portal vein led to a decrease in the collagen type 1-positive area compared with PBS-treated groups. The percentage of the collagen type 1-positive area decreased from 16.02 ± 1.55% to 0.139 ± 0.02% in BM-MSCs-Po (Figure 3(d)).

BM-MSC transplantation (Figures 5(j) and 5(l)) caused a reduction in *α*-SMA-positive cells compared with PBS-treated groups (Figures 5(f) and 5(h)) and the model group (Figure 5(d)). The decrease in *α*-SMA protein expression inferred an improvement in hepatic functions after cell transplantation.

GFP-positive cells were detected at 21 days after transplantation in both treatment groups (Figures 2(e)–2(h)). In BM-MSCs-Ta, BM-MSCs had migrated to the injured liver.

## 4. Discussion

Many stem cell types, such as HSCs [4], BM stem cells [6, 7, 11, 23–25, 27, 33–35], and adipose-derived stem cells [36–40], have been investigated for liver transplantation and treatment of end-stage liver disease (ESLD). Although recent studies have shown positive effects of stem cell therapy in ESLD treatment, there are controversies regarding the source of stem cells and administration strategies. Recent studies have revealed that BM-MSCs can differentiate into hepatic cells* in vitro* [13, 15, 16, 41–43] and induce significant amelioration of liver cirrhosis* in vivo* [4, 9, 21, 44, 45]. In addition to preclinical validation of BM-MSCs, comparisons of various injection routes should be considered for clinical therapies. Therefore, we investigated the influence of cell transplantation routes in a liver cirrhosis model.

In this study, mouse BM-MSCs were isolated and characterized according to previous studies. The results showed that BM-MSCs had a spindle shape, plasticity, and appropriate phenotypes. The BM-MSCs were capable of differentiation into adipogenic cells and bone cells. These results were similar to those in previous studies [8, 31].

CCl_4_ is often used to establish an experimental model of liver cirrhosis. Under the effects of CCl_4_, AST and ALT leak from damaged liver cells. However, liver cells can proliferation to replace the damaged cells after CCl_4_ treatment. This recovery caused a reduction in the AST and ALT levels of PBS-treated groups. Interestingly, AST and ALT levels of BM-MSC-treated groups improved more efficiently than those of PBS-treated and model groups before transplantation. However, there was no significant difference between transplantation of BM-MSCs via the tail or via portal veins. AST and ALT are an indicator of liver injury in case of inflammation and necrosis in liver tissue. Patients with chronic hepatitis or cirrhosis can still have normal aminotransferase levels in multiple tests [46, 47]. In this study, we did not maintain CCl_4_ during treatment that implied reduction of inflammation and liver injury. Similarly, direct bilirubin and albumin levels changed positively in BM-MSC-treated groups. Statistical analysis showed no significant difference in direct bilirubin and albumin levels between BM-MSC transplantation via tail and BM-MSC transplantation via portal veins. These results indicated equivalent therapeutic effects of the two routes on liver injury/liver function markers. Generally, MSCs recover liver function markers at 21 days after transplantation. Our results are in agreement with previous reports [9, 11, 28, 48]. MSCs secrete numerous factors, such as nitric oxide and prostaglandin E2 [2], which enhance antioxidant defenses, inhibit oxidation factors, and reduce necrosis of hepatocytes (Pulavendran et al., 2010) [49]. Furthermore, immune modulation of MSCs inhibits inflammatory cell proliferation, thereby exerting anti-inflammatory effects [6, 7, 13]. We consider that these key functions of BM-MSCs are responsible for the liver injury/function marker recovery after transplantation.

Fibrogenesis-related gene expression of BM-MSC-treated groups included significant downregulation of integrins, procollagen-*α*1, and TGF. TGF plays a major role in stimulation of ECM gene expression in fibroblasts (Gressner et al., 2002) [50]. In our study, the reduction in the fibrogenesis-related gene expression of BM-MSC-treated mice might be related to TGF and HSCs [35, 51]. BM-MSCs inhibit activation of Smad transcription factors [26] that are induced by TGF in HSCs. Similarly, Jang et al. [35] revealed that BM-MSCs reduce TGF-*β*1 and collagen type gene expression to induce recovery of liver fibrosis. In this study, we found significant decreases in collagen type 1 and *α*-SMA protein expression after BM-MSC transplantation, which is consistent with the study of Jang et al. [35]. MSCs secrete enzymes called matrix metalloproteinases (MMPs) (Li et al., 2013) [52]. MMPs play a key role in reorganization of the ECM and digest liver scars or fibrotic fibers (Kang et al., 2012) [53]. Although we did not evaluate MMPs after transplantation, we highly recommend MMPs roles in liver fibrosis treatment. In terms of the presence of BM-MSCs in the injured liver tissue, we observed GFP-positive BM-MSCs at 21 days after transplantation in both BM-MSC-treated groups. It has been revealed that the numbers of MSCs that home to the liver might not be related to the administration route of MSCs [9, 29]. We believe that the timing and numbers of MSCs that home to the liver might be related to the specific circumstances of the liver injury. Although Hong et al. [54] indicated the efficiencies of BMMSC transplantation via portal vein compared to liver injection and other routes, portal vein could cause venous pressure and embolism, which increase liver injury [55]. Furthermore, portal vein transplantation is a difficult procedure to conduct in clinic [28]. Therefore, body intravenous is potential procedure because of effectiveness, safety, and convenience.

Several histological scoring systems have been developed to evaluate inflammation and the stage of fibrosis, such as HAI, Batts and Ludwig, and METAVIR. Among these systems, HAI is a complex system and is therefore often used in clinical trials and research but not in clinical diagnosis. In this study, we employed the HAI system to estimate the stage of fibrosis [56]. The results of the grade and stage of fibrosis strengthened our evaluation of BM-MSC effects on liver function markers as well as fibrotic gene and protein expression. All BM-MSC-treated mice showed reductions in inflammation and the fibrosis stage. Based on these results, BM-MSC transplantation is effective for the treatment of liver fibrosis, which corresponds to previous studies [9, 26, 57, 58]. It has been suggested that BM-MSC transplantation restores liver functions through paracrine mechanisms [9, 59], cell replacement [56], secretion of MMPs, reduced expression of *α*-SMA [22, 57], suppression of liver cell apoptosis, and inhibition of HSCs [60]. However, our comparison of the effectiveness of different injection routes showed no significant difference (*p* > 0.05).

Notably, the portal vein injection group showed significant differences (*p* < 0.05) in procollagen gene expression compared with the tail vein injection group. In contrast, results of liver histology classification showed that tail vein injection group had a lower inflammation grade than the portal vein group. Overall, BM-MSC transplantation via tail or portal veins improves liver cirrhosis disease. Interestingly, there were no differences in the treatment effects between tail and portal vein administrations.

## 5. Conclusions

BM-MSC transplantation ameliorates liver functions in a mouse model of CCl_4_-induced liver fibrosis. At 21 days after cell injection, liver injury markers (AST and ALT) and function markers (bilirubin and albumin) had positive changes compared with untreated groups. Furthermore, BM-MSC transplantation reduced the expression of fibrogenesis- and ECM-related genes and the stage of cirrhosis. The portal vein injection group had significantly different (*p* < 0.05) procollagen gene expression compared with the tail vein injection group. However, liver serum markers and liver histology classification of both groups showed no differences (*p* > 0.05). Considering safety, BM-MSC transfusion via a peripheral vein is a potential method for liver fibrosis treatment.

## Figures and Tables

**Table 1:** Forward and reverse primer pairs used for qRT-PCR to determine fibrosis biomarkers expression.

Primer	Sequence (5′-3′)	Accession number
GAPDH	F: AAGTTGTCATGGATGACC R: TCACCATCTTCCAGGAGC	XM_011241214

Fibronectin	F: ATGTGGACCCCTCCTGATAGT R: GCCCAGTGATTTCAGCAAAGG	NM_001276408.1

Integrin	F: GCCAGGGCTGGTTATACAGA R: TCACAATGGCACACAGGTTT	XM_011248315.1

TGF-beta	F: CTTCAGCTCCACAGAGAAGAACTGC R:CACAATCATGTTGGACAACTGCTCC	NM_011577.1

Procollagen	F: CCTGGACGCCATCAAGGTCTAC R: CCAAGTTCCGGTGTGACTCG	NM_007742.3

**Table 2:** Level of serum markers in groups after 21 days of transplantation.

Group	AST	ALT	Bilirubin	Albumin
Control	47.15 ± 1.12	40.64 ± 2.02	0.145 ± 0.021	1.99 ± 0.057
Model	242.58 ± 12.6	412.18 ± 90.64	0.257 ± 0.022	1.75 ± 0.042
Placebo-Ta	145.6 ± 61.7	255 ± 87.3	0.231 ± 0.032	1.726 ± 0.096
Placebo-Po	218.61 ± 27.71	167.83 ± 18.23	0.315 ± 0.072	1.889 ± 0.048
BM-MSCs-Ta	132.2 ± 11.8	155.8 ± 31.3	0.146 ± 0.033	2.11 ± 0.119
BM-MSCs-Po	178.42 ± 7.34	154.22 ± 6.72	0.128 ± 0.030	2.263 ± 0.126

**Table 3:** Histological grading and staging of chronic hepatitis in experimental groups according to the Knodell-Ishak index (Ishak-modified HAI).

Group	Necroinflammatory scores	Architectural changes, fibrosis, and cirrhosis
Control	1/18	0/6
Model (CCl4)	10/18–15/18	3–5/6
Placebo-Ta	6/18–13/18	1/6 (66.7%), 0/6 (33.3%) (*n* = 10)
BM-MSCs-Ta	2/18–5/18	0/6 (100%) (*n* = 10)
Placebo-Po	4/18–12/18	1/6 (66.7%), 0/6 (33.3%) (*n* = 10)
BM-MSCs-Po	4/18–6/18	0/6 (100%) (*n* = 10)
